# Peripheral S-(PGJ_2_)-glutathione as a diagnostic biomarker for depression-anxiety comorbidity: development and validation

**DOI:** 10.1186/s12888-026-08276-7

**Published:** 2026-06-11

**Authors:** Yan Zhen, Yuhao Gan, Xigao Liu, Jun Li, Shihao Wei, Zelin Li, Jin Hu, Chengtao Nie, Fan Wu, Yiyi Liu, Zezhi Li, Zhengyi Luo

**Affiliations:** 1 The Affiliated Brain Hospital, Guangzhou Medical University, Guangzhou, 510370 China; 2 Guangdong Engineering Technology Research Center for Translational Medicine of Mental Disorders, The Affiliated Brain Hospital, Guangzhou Medical University, Guangzhou, 510370 China; 3 School of Mental Health, Guangzhou Medical University, Guangzhou, 510370 China; 4 Department of Urology, Qilu Hospital, Cheeloo College of Medicine, Shan Dong University, Jinan, 250012 China; 5 Department of Laboratory Medicine, Zhujiang Hospital, Southern Medical University, Guangzhou, 510282 China; 6 Guangzhou Institute of Pediatrics, Guangzhou Women and Children’s Medical Center, State Key Laboratory of Respiratory Diseases, Guangzhou Medical University, Guangzhou, 510623 China; 7 Guangdong Provincial Key Laboratory of Psychiatric Disorders, Southern Medical University, Guangzhou, 510515 China

**Keywords:** S-(PGJ_2_)-glutathione, Prostaglandins, Glutathione conjugates, Comorbid depression and anxiety disorders, Diagnostic marker

## Abstract

**Background:**

Comorbidity of depression and anxiety disorders (DAs) is as high as 50%, and diagnosis remains heavily reliant on subjective symptomatic assessments due to the lack of validated objective biomarkers. Neuroinflammation and oxidative stress are well-recognized core pathophysiological features of DAs. Prostaglandins (PGs), a class of lipid mediators closely linked to neuroinflammation and oxidative stress, have been implicated as key mediators in the pathogenesis of mood and anxiety disorders. S-(PGJ₂)-glutathione, a covalent conjugate of 15d-PGJ₂ and glutathione (GSH), integrates PG-mediated inflammatory signaling and GSH-dependent antioxidant defense, suggesting its potential as a candidate biomarker for DAs.

**Methods:**

The case-control study enrolled 77 participants, including 39 patients with comorbid depression and anxiety disorders (DAs) and 38 healthy controls (HCs) matched for gender, age, and body mass index (BMI). The cohort was randomly stratified into training and test sets at a 7:3 ratio. Serum levels of PG-related metabolites were quantified using liquid chromatography-tandem mass spectrometry (LC-MS/MS). Univariate and multivariate logistic regression analyses were performed in the training set to identify independent biomarkers. Receiver operating characteristic (ROC) analysis was employed to assess diagnostic performance in the training cohort, test cohort, and overall population, while decision curve analysis (DCA) was used to evaluate clinical utility.

**Results:**

A total of 21 PG-related metabolites were detected, of which five were significantly dysregulated in DAs patients and remained significant after FDR correction for multiple testing. Multivariate logistic regression identified S-(PGJ₂)-glutathione as an independent biomarker associated with DAs, both before and after adjustment for confounding factors including education level, systolic blood pressure (SBP), and diastolic blood pressure (DBP). ROC analysis in the total cohort showed that S-(PGJ₂)-glutathione yielded an AUC of 0.949, with a sensitivity of 0.789 and specificity of 0.949. Consistent results were observed in the training and internal test sets. DCA suggested that using S-(PGJ₂)-glutathione for diagnosis may provide a higher net benefit than conventional “Treat All” or “Treat None” strategies over a wide range of threshold probabilities.

**Conclusion:**

The PG metabolic pathway is dysregulated in patients with DAs. S-(PGJ₂)-glutathione is significantly downregulated and exhibits favorable preliminary diagnostic efficacy based on internal training and test set validation. Given the relatively small sample size and the absence of external cohort validation, these findings should be interpreted as preliminary.

## Introduction

Depression and anxiety disorders, two of the most common mental illnesses globally, impose a substantial burden on both individuals and healthcare systems, with a comorbidity rate of approximately 50% [1]. Comorbid cases are associated with more severe clinical symptoms, greater functional impairment, longer disease duration, and poorer treatment outcomes compared to either disorder alone [2–4]. Despite their high prevalence and clinical significance, the diagnosis of DAs remains a major challenge in clinical practice, largely due to the significant symptomatic overlap between depression and anxiety, and the lack of reliable, objective biological markers [1]. Current diagnostic approaches depend heavily on subjective clinical evaluations and self-reported symptoms, which may not fully capture the underlying biological complexity of these disorders [1, 5, 6]. Thus, there is an urgent need to identify quantifiable, disease-specific biomarkers that can improve diagnostic accuracy, facilitate early detection, and ultimately guide personalized treatment strategies for DAs.

To address this unmet clinical need, accumulating studies have explored potential diagnostic biomarkers for DAs in blood and urine. For example, a urinary metabolite panel has been proposed as a candidate diagnostic tool for depression and anxiety disorders [1], while inflammatory markers such as C-reactive protein (CRP) have been implicated in the pathophysiology of comorbid cases [7]. Peptide-based biomarkers for DAs are also under active investigation. Given the complex pathophysiology of DAs, biomarkers reflecting a single pathway may not fully capture disease heterogeneity. This underscores the need to explore novel biomarkers that integrate multiple key pathogenic mechanisms to improve diagnostic accuracy and clinical applicability.

In recent years, accumulating evidence has highlighted that neuroinflammation and oxidative stress are core, interconnected pathophysiological mechanisms underlying the development and progression of depression and anxiety disorders [6, 8–11]. Neuroinflammation is characterized by increased activation of microglia and astrocytes, along with elevated levels of pro-inflammatory cytokines (e.g., interleukin-6, tumor necrosis factor-α) in the central nervous system (CNS) [12–14]. These pro-inflammatory mediators disrupt the function of mood-regulating neural circuits, including the hippocampus, amygdala, and prefrontal cortex, and impair monoamine neurotransmission (e.g., serotonin, dopamine) and neuroplasticity, key processes implicated in the pathogenesis of mood disorders [6]. Oxidative stress, conversely, is characterized by an imbalance between reactive oxygen species (ROS) production and the ability of the antioxidant defense system to counteract them [15, 16]. Persistent oxidative stress leads to oxidative damage to proteins, lipids, and DNA in neuronal cells, further exacerbating neuronal dysfunction and contributing to the progression of DAs [17]. Notably, neuroinflammation and oxidative stress are mutually reinforcing in DAs: pro-inflammatory cytokines promote ROS production, while oxidative stress enhances microglial activation and pro-inflammatory cytokine release, forming a vicious cycle that perpetuates neuronal injury in DAs.

Prostaglandins (PGs), a class of twenty-carbon unsaturated fatty acid derivatives synthesized from arachidonic acid via cyclooxygenase (COX-1/2) catalysis, are key regulators of both neuroinflammation and oxidative stress [18]. Unlike systemic hormones, PGs act as local mediators with a short plasma half-life (only 1–2 min) and are rapidly inactivated, allowing for precise, time-limited regulation of inflammatory and redox responses [19]. Specific PG subtypes, including PGE, PGD₂, and PGJ₂, have been specifically implicated in the regulation of CNS inflammatory processes and mood-related behaviors [20–22]. Among these, PGJ₂, derived from the dehydration of PGD₂, functions as a potent pro-inflammatory and pro-oxidant mediator [23]. PGJ₂ undergoes subsequent dehydration to yield 15-deoxy-Δ¹²,¹⁴-prostaglandin J₂ (15d-PGJ₂), one of the most reactive cyclopentenone prostaglandins [24]. S-(PGJ₂)-glutathione is mainly formed by the covalent conjugation of 15d-PGJ₂ with glutathione (GSH). For simplicity, we use the term S-(PGJ₂)-glutathione consistently throughout the manuscript. PGJ₂ has been shown to promote the accumulation of ubiquitinated proteins, induce neuronal apoptosis, and exacerbate oxidative stress, with implications in various neurodegenerative pathologies such as Alzheimer’s disease and Parkinson’s disease [22, 23, 25]. Given its role in neuroinflammation and oxidative stress, two core mechanisms of DAs, PGJ₂ is likely to be involved in the pathophysiology of comorbid depression and anxiety, though its specific role remains incompletely understood.

Glutathione (GSH), the major intracellular antioxidant, is essential for maintaining redox homeostasis and safeguarding cells from oxidative damage [26]. GSH deficiency is a consistent finding in patients with depression and anxiety disorders, accompanied by reduced activity of key antioxidant enzymes (e.g., glutathione peroxidase, glutathione reductase) [27–29]. This impairment in the antioxidant defense system leads to ROS accumulation, further amplifying oxidative stress and neuroinflammation in DAs [8]. Glutathione conjugates are formed when GSH binds to endogenous or exogenous compounds (e.g., reactive metabolites, toxins, drugs) via catalysis by glutathione-S-transferases (GSTs) [30, 31], a family of enzymes involved in detoxification, metabolic regulation, and cellular signaling [32, 33]. Among these conjugates, S-(PGJ₂)-glutathione is a specific metabolite produced through GSTP1-mediated covalent binding of PGJ₂ to GSH [34]. As such, S-(PGJ₂)-glutathione integrates PGJ₂-mediated inflammatory signaling and GSH-dependent antioxidant defense, making it a potential link between the neuroinflammatory and oxidative stress pathways in DAs.

Although prostaglandins (PGs) and glutathione (GSH) have been reported to exhibit significant alterations in anxiety and depression [28, 29, 35–37], few studies have identified them as reliable biomarkers, mainly due to their poor stability and short half-life [19, 38]. Specifically, GSH is prone to oxidation in vitro and has a plasma half-life of only several minutes [38], while PGs (especially 15-deoxy-Δ12,14-prostaglandin J₂, 15d-PGJ₂) are highly electrophilic and rapidly degrade or bind to biomacromolecules in biological samples, posing challenges for accurate detection [22]. In contrast, S-(PGJ₂)-glutathione, a conjugate of 15d-PGJ₂ and GSH, exhibits improved stability, with its half-life extended to several hours [22, 38]. More importantly, this peripheral blood-derived conjugate may reflect the pathological features of both inflammatory and oxidative stress pathways involved in anxiety and depression, as it integrates the biological implications of PGs (neuroinflammation regulation) and GSH (endogenous antioxidant defense). Its accessibility in peripheral blood also enhances diagnostic operability, endowing it with potentially greater clinical translation potential compared to free PGs and GSH.

Although neuroinflammation, oxidative stress, PG metabolism, and GSH homeostasis are known to be involved in DAs, the status of S-(PGJ₂)-glutathione in this context is unknown. Specifically, its serum levels and diagnostic utility in patients with DAs have not been investigated. Therefore, this study aimed to determine the levels of PG-related metabolites, especially S-(PGJ₂)-glutathione, in these patients and to explore its value as a novel biomarker.

## Materials and methods

### Participants

The participants were from the same clinical cohort as our previous work [39], ensuring consistency in the study population and baseline data collection. Detailed information regarding the study design, ethical approval (Approval Number: 2019-016, Ethics Committee of the Affiliated Brain Hospital of Guangzhou Medical University), participant recruitment procedures, diagnostic criteria, and inclusion/exclusion criteria has been comprehensively described in that manuscript. Briefly, this cohort consisted of 39 patients with comorbid depression and anxiety disorders (DAs) and 38 healthy controls (HCs), all participants signed written informed consent before enrollment. Newly diagnosed patients with DAs, aged 18–65 years, were included if they met the DSM-5 criteria for both major depressive disorder and generalized anxiety disorder, as confirmed by a structured clinical interview. All participants had a Hamilton Depression Rating Scale (HAMD) score > 7 and a Hamilton Anxiety Rating Scale (HAMA) score > 14 [40, 41]. Healthy controls had no current or lifetime history of any major psychiatric disorder (confirmed by structured clinical interview) and were frequency-matched to DA patients by sex, age, and body mass index (BMI) to minimize potential confounding.

Exclusion criteria for all participants included: (1) a diagnosis of other major psychiatric disorders (e.g., obsessive-compulsive disorder, schizophrenia, bipolar disorder); (2) any prior or current use of antidepressant, anxiolytic, antipsychotic, mood-stabilizing, or other psychotropic medications; (3) significant uncontrolled medical conditions (including severe cardiovascular, hepatic, renal, neurological, gastrointestinal, endocrine, or rheumatic diseases, or infectious diseases) that could interfere with the study; (4) a history of epilepsy or other central nervous system disorders; (5) alcohol or substance abuse or dependence within the past 6 months; (6) pregnancy or lactation; (7) inability to comply with study procedures or provide a blood sample.

The present study builds upon the same untargeted metabolomics dataset but focuses on a distinct metabolite panel and investigates novel research hypotheses unrelated to prior analyses.

### Two experimental sets

Two experimental subsets (training set and test set) were constructed to preliminarily assess the internal consistency of the study results. All enrolled patients with depression and anxiety disorders and healthy controls (HCs) were stratified and randomly divided into the training and test set at a ratio of 7:3, with stratification factors including gender, age, and BMI to maintain the consistency of baseline characteristics between the two subsets.

After random allocation, the training set contained 27 patients and 27 HCs, whereas the test set contained 12 patients and 11 HCs. The training set was primarily used to screen for differentially expressed metabolites and to identify potential diagnostic biomarkers, which laid the foundation for subsequent diagnostic performance evaluation. The test set, which was completely independent of the training set, was exclusively used to validate the diagnostic efficacy of the candidate biomarker in an unbiased manner.

### Blood collection

All participants provided blood samples in the morning following an overnight fast of at least 12 h. Before collection, all participants were advised to avoid strenuous physical activity, alcohol consumption, caffeine intake, and smoking for 24 h. A 2 mL venous blood sample was drawn into sterile vacuum blood tubes without anticoagulants. Tubes were gently inverted 3 to 5 times immediately after collection and kept upright at room temperature (22–25 °C) for natural clotting for 1 h. Serum was collected after centrifugation at 3,000 rpm for 10 min at 4 °C and then transferred into 1.5 mL RNase-/DNase-free polypropylene microcentrifuge tubes. Serum was immediately divided into 200 µL aliquots to avoid repeated freeze-thaw cycles and stored at -80 °C for subsequent LC-MS/MS analysis. All sample processing procedures were finished within 2 h of blood collection.

### Metabolomic analysis

Serum was deproteinized using a methanol-acetonitrile solution and subsequently analyzed by LC-MS/MS (OE Biotechnology Co., Ltd., Shanghai, China.). An ACQUITY UPLC HSS T3 column (100 mm × 2.1 mm, 1.8 μm; maintained at 45 °C) was used for chromatographic separation on a Waters ACQUITY UPLC I-Class Plus system coupled to a Thermo QE HF mass spectrometer (Thermo Fisher Scientific, Germany). The mobile phase consisted of 0.1% formic acid in water (A) and acetonitrile (B), delivered at a flow rate of 0.35 mL/min with the following gradient program: 95% A (0–2 min), 70% A (2–4 min), 50% A (4–8 min), 20% A (8–10 min), 100% B (10–15 min), and 95% A (15.1–16 min). Mass spectrometric detection was carried out using a Thermo QE HF instrument equipped with an electrospray ionization (ESI) source operating in both positive and negative ionization modes.

### Statistical analysis

All statistical analyses were conducted with SPSS 22. All reported P values are two-sided, and *p* < 0.05 was considered statistically significant. Categorical data were assessed by the Chi-square test, and continuous data were evaluated using the Mann–Whitney U test. Missing values were imputed with the respective limits of detection. Binary logistic regression was used to determine odds ratios (OR) and 95% confidence interval (CI) of S-(PGJ₂)-glutathione in depression and anxiety disorders. The diagnostic performance of S-(PGJ_2_)-glutathione was evaluated by receiver operating characteristic (ROC) curve analysis, with the area under the curve (AUC) reported. The 95% CIs for the AUC were calculated using the bootstrap method with 1,000 resamples. DCA was used to assess the clinical utility of S-(PGJ_2_)-glutathione in disease. Data are shown as mean ± standard deviation (SD) with *p* < 0.05.

## Results

### Characterization of the study population

This study included 77 participants, comprising 39 patients with depression and anxiety disorders and 38 healthy controls. The two groups were matched for gender, age, and BMI. The entire cohort was randomly stratified into training and test sets at a 7:3 ratio, with stratification by gender, age, and BMI to ensure balanced baseline characteristics between the two subsets. Significant between-group differences in systolic blood pressure (SBP), diastolic blood pressure (DBP), HAMA and HAMD scores were observed in both the total cohort and training set. Educational level differed significantly in the total cohort, with a borderline significance in the training set (*p* = 0.0640). The baseline characteristics of the study population are summarized in Table 1.

**Table 1:** Demographic and clinical characteristics

Variable	Total cohort			Training Set			Test Set		
	HC (*N* = 38)	DA (*N* = 39)	*p*	HC (*N* = 27)	DA (*N* = 27)	*p*	HC (*N* = 11)	DA (*N* = 12)	*p*
Gender (male/female)	20/18	21/18	0.915	15/12	15/12	1.000	5/6	6/6	0.827
Age	27.58 ± 7.236	26.67 ± 8.640	0.2449	26.59 ± 6.687	26.41 ± 8.372	0.3232	30.00 ± 8.270	27.25 ± 9.574	0.3712
BMI	21.31 ± 2.600	22.98 ± 5.872	0.2480	21.32 ± 2.707	22.65 ± 4.914	0.3713	21.37 ± 2.789	23.43 ± 7.835	0.8328
Education	15.32 ± 2.951	14.15 ± 2.916	0.0358	16.00 ± 2.130	14.63 ± 2.924	0.0640	15.00 ± 2.145	13.08 ± 2.712	0.1004
SBP	109.40 ± 12.13	118.20 ± 18.20	0.0168	107.8 ± 11.03	118.1 ± 14.46	0.0033	113.2 ± 14.33	118.4 ± 24.88	0.7276
DBP	70.37 ± 7.872	74.70 ± 8.359	0.0225	69.48 ± 7.861	75.04 ± 7.434	0.0108	72.55 ± 7.828	74.32 ± 10.42	0.5538
HAMA	2.579 ± 1.130	24.85 ± 5.842	< 0.0001	2.667 ± 1.177	26.26 ± 5.933	< 0.0001	2.364 ± 1.027	21.67 ± 4.334	< 0.0001
HAMD	2.079 ± 0.6317	23.15 ± 4.755	< 0.0001	2.037 ± 0.5871	23.52 ± 4.941	< 0.0001	2.182 ± 0.7508	22.33 ± 4.397	< 0.0001

Continuous variables are presented as mean ± SD and were compared using the Mann–Whitney U test (normality not assumed). Categorical variables were compared using the chi-square test

### Differentially expressed PG-related metabolites between two groups

**Fig. 1:**
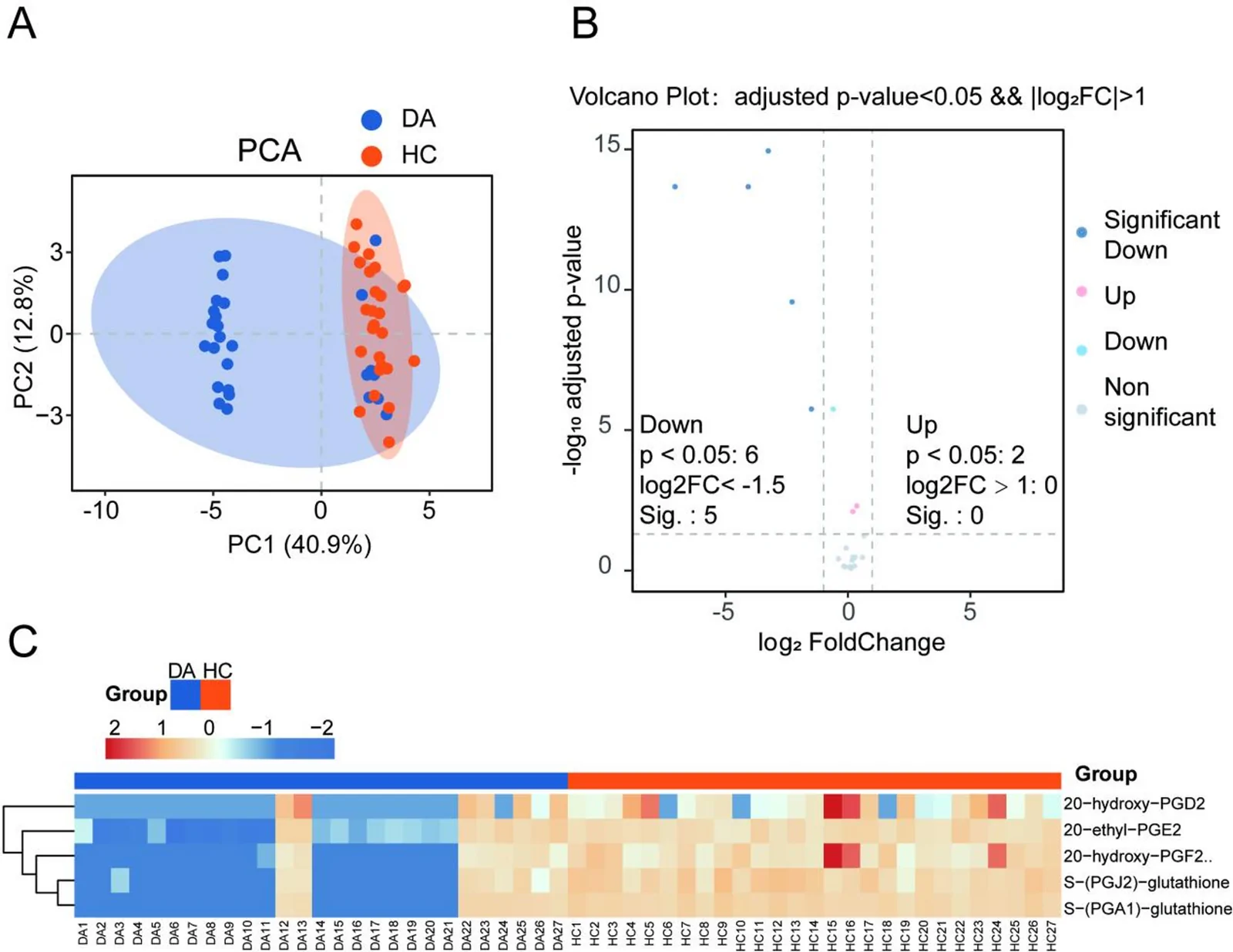
Differentially expressed PG-related metabolites between two groups. **(A)** PCA shows a trend toward separation between the two groups. **(B)** The volcanic map shows the differential expressed PG-related metabolites. The x-axis shows log2 fold change between DA patients and healthy controls. The y-axis shows -log10(adjusted *p* value) after false discovery rate (FDR) correction across all 21 tested metabolites. The horizontal dashed line indicates adjusted *p* = 0.05. Five metabolites met this criterion, all of which were downregulated. **(C)** Hierarchical clustering heatmap of the five PG-related metabolites that showed significant differential expression after FDR correction across all 21 tested metabolites (adjusted *p* < 0.05). Each column represents a participant, and each row represents a metabolite. Red represents high level and blue indicates low level. HC: health control; DA: depression and anxiety disorders

The levels of PG-related metabolites were quantified using LC-MS/MS. Principal component analysis (PCA) showed a trend toward separation between the two groups, suggesting that the overall metabolomic profiles differed but with some overlap (Fig. 1A). To identify differentially expressed metabolites, fold change (FC) > 1 was used as a cutoff. Given that 21 PG-related metabolites were detected, the false discovery rate (FDR) was controlled using the Benjamini–Hochberg procedure to adjust for multiple comparisons. Both the unadjusted *p*-values and FDR- adjusted *p*-values for the 21 metabolites are listed in Table 2. Metabolites with an adjusted *p* < 0.05 and FC > 1 were considered statistically significant. Under this criterion, five metabolites were significantly downregulated.

**Table 2:** The original and adjusted *p*-values for 21 PG-related metabolites in the training set

Variable	Original *p*	Adjusted *p*
S-(PGJ2)-glutathione	5.438E-17	1.142E-15
20-hydroxy-PGF2α	3.027E-15	2.150E-14
S-(PGA1)-glutathione	3.071E-15	2.150E-14
20-ethyl-PGE2	5.179E-11	2.719E-10
20-hydroxy-PGD2	4.249E-07	1.770E-6
Prostaglandin F-main urinary metabolite	5.057E-07	1.770E-6
11-deoxy-11-methylene-PGD2	1.655E-03	4.966E-03
11-deoxy-16,16-dimethyl-PGE2	3.026E-03	7.943E-03
Prostaglandin E2 ethanolamide	0.02482	0.05792
Dhv-PGE2	0.07439	0.15623
15-deoxy-delta-12,14-PGJ2	0.17440	0.33294
PGF2α-dihydroxypropanylamine	0.19784	0.33528
PGE3 1,15-lactone	0.20755	0.33528
6-keto PGE1	0.25302	0.37954
11-deoxy-11-methylene-15-keto-PGD2	0.30888	0.43243
PGE1 alcohol	0.53748	0.68728
PGF2α dimethyl amide	0.55637	0.68728
PGH1	0.6605	0.74134
13,14-dihydro-15-keto-PGA2	0.70297	0.74134
19-Hydroxyprostaglandin E1	0.70604	0.74134
11-deoxy-PGF2a	0.84000	0.84001

The volcano plot (Fig. 1B) and hierarchical clustering heatmap (Fig. 1C) were generated based on the adjusted *p*-values and illustrate the separation of the five metabolites between the two groups. Collectively, these results suggest that candidate metabolites were consistently identified and support the statistical validity of our findings within this cohort.

### Logistic regression analysis of S-(PGJ₂)-glutathione predicting depression and anxiety disorders

A multivariate binary logistic regression analysis was performed in the training set to identify PG-related metabolites independently associated with disease status (diag: 0 = control, 1 = case), using the five metabolites as candidate variables. Stepwise selection was used for variable selection, with collinearity diagnostics and residual analysis performed to validate the model. Only S-(PGJ₂)-glutathione was retained in the final stepwise model, and was significantly associated with disease status (OR = 0.057; 95% CI: 0.005–0.647; *p* = 0.021) (Table 3). These results suggest that S-(PGJ₂)-glutathione may be a potential biomarker to differentiate patients from healthy controls in the current study.

**Table 3:** Logistic regression analysis of 5 PG-related metabolites in the training set

Variable	β	OR	*p*	95%CI
S-(PGJ2)-glutathione	-2.859	0.057	0.021	0.005–0.647

Additionally, several covariates, including education level, systolic blood pressure (SBP), and diastolic blood pressure (DBP), were identified as potential confounders because all showed *p* < 0.1 in univariate analyses. These covariates were incorporated into the adjusted multivariate model based on statistical criteria and their established clinical relevance to mental health outcomes [42–45]. After adjustment for these confounding factors, multivariate analysis showed that S-(PGJ₂)-glutathione remained an independent predictor of depression and anxiety disorders (adjusted OR = 0.010; 95% CI, 0.000–0.451; *p* = 0.018). The univariate and adjusted multivariate regression results for the target metabolite and all covariates are summarized in Table 4.

**Table 4:** Univariate and multivariate logistic regression analyses of S-(PGJ2)-glutathione in depression and anxiety disorders in the training set

Variable	OR (95%CI)	*p*	OR (95%CI)	*p*
S-(PGJ2)-glutathione	0.057(0.005–0.647)	0.021	0.010(0.000-0.451)	0.018
gender	1.000(0.342–2.926)	1.000		
age	0.997(0.928–1.071)	0.927		
BMI	1.097(0.943–1.277)	0.230		
education	0.805(0.641–1.010)	0.061	0.608(0.324–1.138)	0.120
SBP	1.067(1.016–1.121)	0.010	1.026(0.895–1.176)	0.712
DBP	1.099(1.019–1.184)	0.014	1.072(0.852–1.350)	0.553

### ROC analysis

**Fig. 2:**
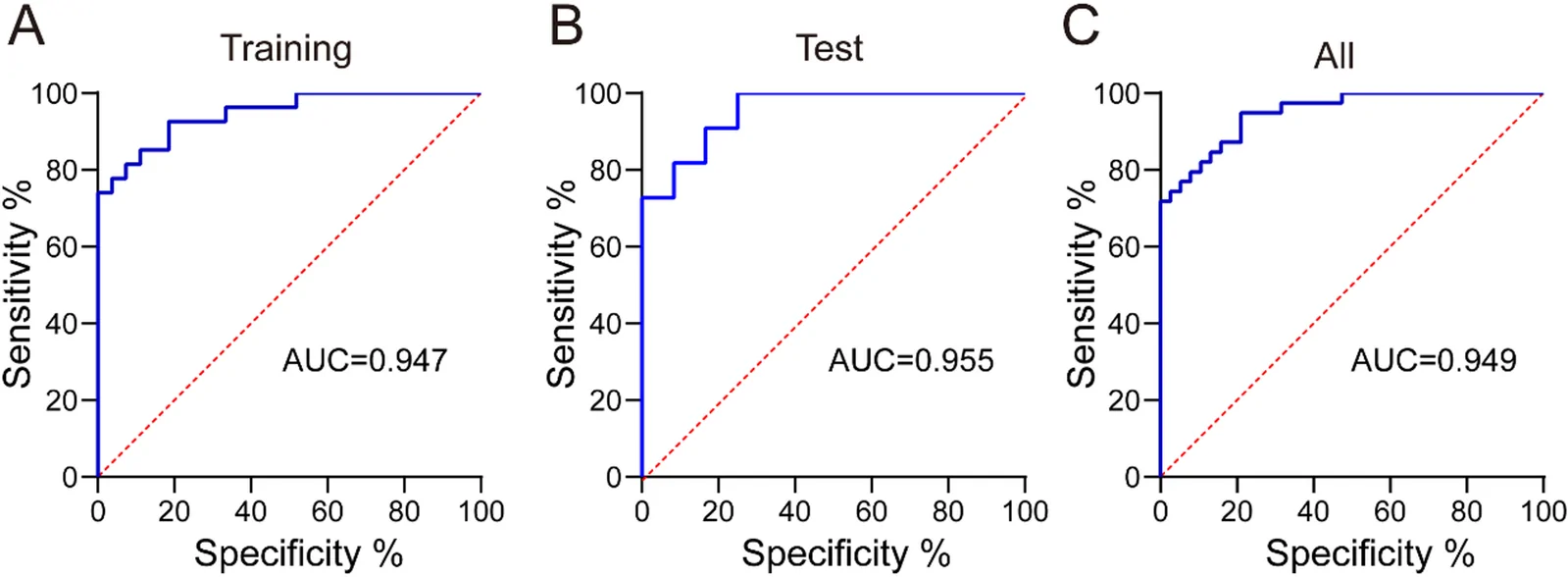
The ROC curve for the diagnostic marker S-(PGJ₂)-glutathione of depression and anxiety disorders. (**A**-**C**) The AUC of S-(PGJ₂)-glutathione was 0.947, 0.955 and 0.949 in the training, test and all set, respectively

ROC analysis was conducted to assess the diagnostic potential of S-(PGJ₂)-glutathione for depression and anxiety disorders. In the training set, S-(PGJ₂)-glutathione showed an ability to independently distinguish patients from HCs, with an AUC of 0.947 (Fig. 2A). At the optimal cutoff value of 11.315, this metabolite showed a sensitivity of 0.815 and a specificity of 0.926 (Table 5). Consistently, in the internal test set, S-(PGJ₂)-glutathione also showed discriminatory capacity between patients and HCs, with an AUC of 0.955 (Fig. 2B). At the optimal cutoff value of 8.257, the sensitivity and specificity of S-(PGJ₂)-glutathione were 1 and 0.750, respectively (Table 5). In the total cohort, S-(PGJ₂)-glutathione also showed an ability to independently differentiate patients from HCs, with an AUC of 0.949 (Fig. 2C). At the optimal cutoff value of 11.326, the sensitivity and specificity were 0.789 and 0.949, respectively (Table 5). Collectively, these findings suggest that, pending external validation, S-(PGJ₂)-glutathione may be a potential diagnostic biomarker for depression and anxiety disorders.

**Table 5:** The diagnostic performance of S-(PGJ2)-glutathione for depression and anxiety disorders

Variable	Set	AUC	95% CIs	Cutoff Value	Sensitivity	Specificity	*p*
S-(PGJ2)-glutathione	Training	0.947	0.879–0.990	11.315	0.815	0.926	< 0.001
	Test	0.955	0.861-1.000	8.257	1	0.750	< 0.001
	Total	0.949	0.907–0.992	11.326	0.789	0.949	< 0.001

95% CIs were calculated using the bootstrap method with 1,000 resamples

### DCA analysis

**Fig. 3:**
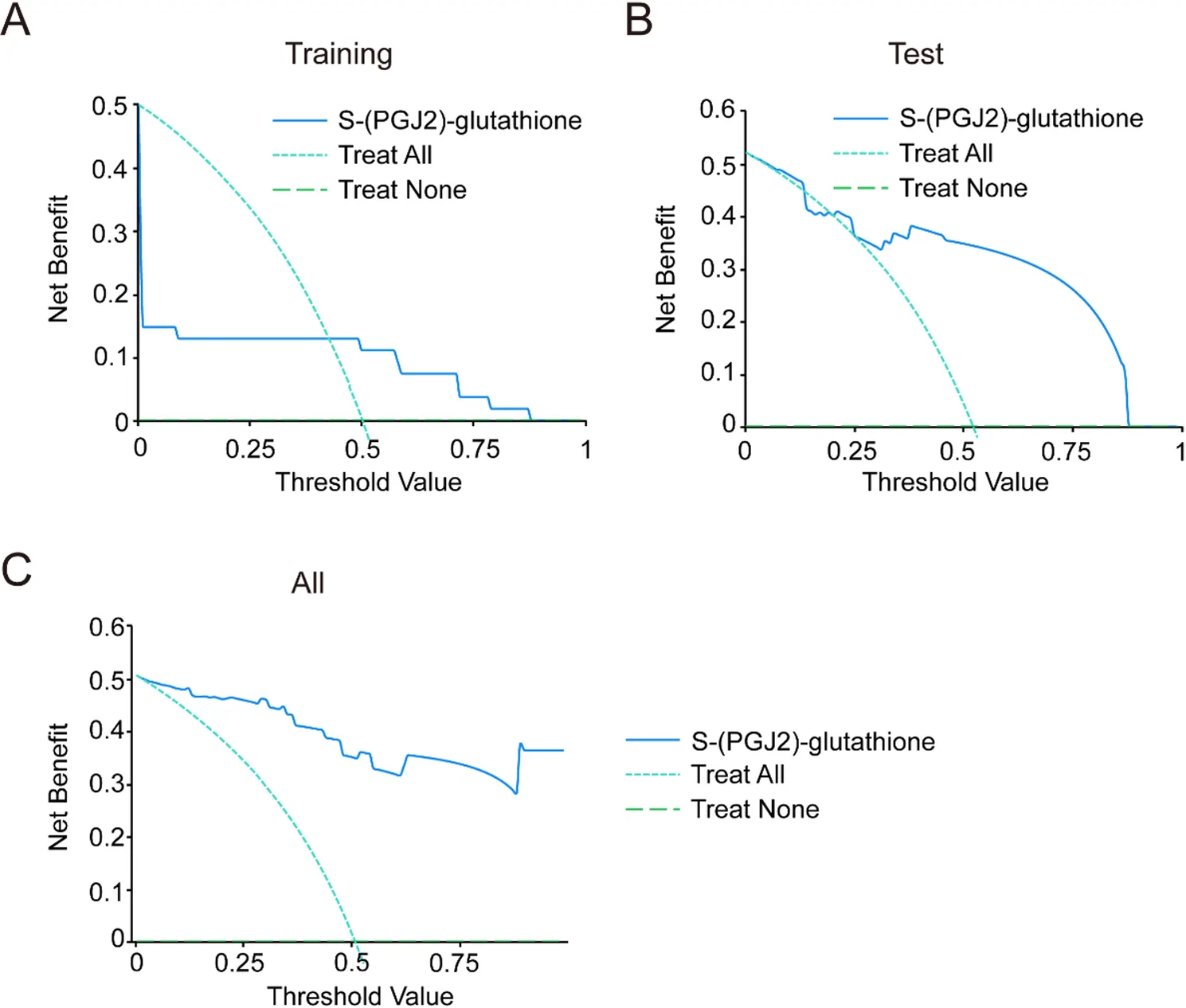
Decision curve analysis (DCA) of S-(PGJ₂)-glutathione for diagnosing depression-anxiety comorbidity. **(A-C)** DCA results for the training set, test set, and total cohort, respectively. The y-axis indicates the net clinical benefit, and the x-axis represents the threshold probability of clinical decision-making. The horizontal green long-dash line corresponds to the “Treat None” strategy, while the green dotted-dash line represents the “Treat All” strategy. The blue curve denotes the net clinical benefit of applying S-(PGJ₂)-glutathione for diagnostic decision-making. When the biomarker curve lies above the two green reference lines, the biomarker provides additional clinical net benefit within a certain range of threshold probabilities. The biomarker showed clinical utility when the threshold probability exceeded 0.43 in the training set, 0.25 in the test set, and 0.02 in the total cohort

DCA was conducted to assess the clinical utility of S-(PGJ₂)-glutathione (Fig. 3A-C). In the training set, DCA suggested that using the metabolite was associated with a greater net benefit than the “Treat All” and “Treat None” strategies when the threshold probability of disease was greater than 0.43. In the test set, S-(PGJ₂)-glutathione provided a greater net benefit than the two reference strategies at threshold probabilities above 0.25. In the total cohort, the metabolite yielded a higher net benefit than both “Treat All” and “Treat None” strategies when the threshold probability exceeded 0.02. Together, these findings suggest that S-(PGJ₂)-glutathione may provide favorable clinical utility for the identification of patients with depression and anxiety disorders in this cohort.

## Discussion

This study explored the diagnostic potential of PG-related metabolites in comorbid depression and anxiety disorders (DAs), identifying S-(PGJ₂)-glutathione as a promising serum biomarker. The glutathione conjugate of 15d-PGJ₂ is significantly downregulated in DAs patients and exhibits favorable preliminary diagnostic performance (AUC = 0.949) with consistent internal stratified validation results. Notably, these preliminary findings are limited by the small sample size and lack of external independent verification, and thus should be interpreted cautiously.

Oxidative-antioxidative imbalance is a well-established pathophysiological feature of anxiety and depression, and accumulating studies have explored potential diagnostic biomarkers for DAs in blood and urine to address the lack of objective diagnostic tools. For instance, a urinary biomarker panel comprising aminomalonic acid, N-methylnicotinamide, hippuric acid, and azelaic acid has been proposed for the potential diagnosis of depression and anxiety disorders [1]. Similarly, C-reactive protein (CRP), an inflammatory marker, has been implicated in the pathophysiology of DAs [7]. Furthermore, our previous research has identified short peptide biomarkers with diagnostic value for DAs [39]. These findings further expand the panel of candidate biomarkers for this comorbid disorder. Oxidative stress dysregulation is well documented in DAs. A previous study including 101 patients with generalized anxiety disorder reported increased serum malondialdehyde (MDA), lipid hydroperoxides (LPO) and cortisol, together with decreased glutathione peroxidase (GSH-Px), superoxide dismutase (SOD) and catalase (CAT). Baseline MDA also exhibited satisfactory predictive performance for selective serotonin reuptake inhibitors (SSRI) treatment response, with an AUC of 0.804 [37]. However, most existing biomarkers for DAs focus on single pathophysiological pathways, and their clinical utility remains to be established, highlighting the need for novel biomarkers that integrate key pathogenic mechanisms to enhance diagnostic performance. In the current internal cohort, the AUC of S-(PGJ₂)-glutathione (0.949) compared favorably with conventional single markers (e.g., MDA, AUC = 0.804; stromal cell-derived factor-4, AUC = 0.870 [46]) and was similar to NADPH oxidase 1 (NOX1, AUC = 1 [47]) in GAD patients. This favorable performance may be tentatively attributed to its unique role as a molecular intersection of PG-mediated inflammatory signaling and GSH-dependent antioxidant defense, potentially capturing synergistic pathophysiological information that single markers may not capture.

PGJ₂, a cyclopentenone prostaglandin involved in inflammation resolution, and GSH, the brain’s primary endogenous antioxidant, are both implicated in psychiatric disorders [37, 48]. The downregulation of S-(PGJ₂)-glutathione in DAs may reflect enhanced consumption due to chronic oxidative stress, impaired biosynthesis, or altered PG metabolism. This finding aligns with prior observations of elevated oxidative damage markers (e.g., MDA, 8-OHdG [49]) in anxiety disorders, suggesting concurrent dysregulation of protective conjugates and damage markers in DAs. Animal models have shown that chronic stress induces oxidative damage and that antioxidant interventions ameliorate depressive- and anxiety-like behaviors [37], further supporting the relevance of this pathway.

The diagnostic performance of S-(PGJ₂)-glutathione (sensitivity 0.789, specificity 0.949 in the total cohort) presents certain favorable features compared with conventional markers, particularly its high specificity, which may reflect closer alignment with the pathophysiology of comorbid DAs. Notably, most previous studies focused on “pure” anxiety or depression, whereas our findings target the comorbid phenotype–an important distinction given evidence that comorbid presentations may exhibit enhanced nitro-oxidative stress [37]. Compared to existing candidates (e.g., the urinary metabolite panel [1], CRP [7], and our team’s short peptide markers), S-(PGJ₂)-glutathione integrates inflammatory and oxidative stress pathways, complementing these existing biomarkers and suggesting the potential value of multi-pathway targets for DAs diagnosis. However, it remains unclear whether S-(PGJ₂)-glutathione is specific to DAs or a transdiagnostic marker of affective pathology, as oxidative stress alterations are known to overlap across psychiatric disorders [48]. Moreover, the marker’s performance in differentiating comorbidity from “pure” major depression or generalized anxiety disorder has not been tested and will require head-to-head comparison studies.

Decision curve analysis suggested that S-(PGJ₂)-glutathione may provide net clinical benefit over a wide range of threshold probabilities (> 0.02 in the total cohort), offering preliminary support for its potential utility for screening and diagnosis.

### Limitations and future directions

Several key limitations of this study must be acknowledged: First, despite adjusting for education, SBP, and DBP, residual confounding from unmeasured variables, including dietary habits, medication history, physical activity levels, smoking status, and metabolic parameters (e.g., fasting glucose, lipid profiles), cannot be excluded, which may have influenced the metabolite levels and study outcomes. Second, the absence of psychiatric control groups (e.g., major depressive disorder-only, generalized anxiety disorder-only, or other anxiety disorders) precludes assessment of whether S-(PGJ₂)-glutathione specifically identifies the comorbid DAs phenotype or reflects general psychiatric distress. Third, the cross-sectional study design provides no information on the temporal stability of S-(PGJ₂)-glutathione levels, nor can it address whether the metabolite normalizes with effective treatment or predicts treatment response. Fourth, the small overall sample size, particularly the limited size of the test cohort, may raise concerns regarding potential overfitting. Although the internal training/test split provided initial evidence of internal consistency, it cannot replace external validation. Future studies with larger independent cohorts are required to confirm the stability of the AUC estimates. Nevertheless, the consistent AUC values observed in the training (0.947), test (0.955), and total cohorts (0.949) suggest that the favorable diagnostic performance is unlikely to be driven by random chance or overfitting bias within this dataset. While the current findings suggest that S-(PGJ₂)-glutathione may be a promising candidate marker for the comorbid DAs phenotype, its performance in differentiating comorbidity from ‘pure’ major depression or generalized anxiety remains an open, clinically vital question to be addressed in subsequent head-to-head comparison studies. Finally, we cannot rule out the possibility that changes in S-(PGJ₂)-glutathione may also be observed in other psychiatric disorders or somatic diseases associated with oxidative stress and inflammation, which warrants further investigation in heterogeneous clinical populations.

Future research should prioritize external validation in large, multi-center cohorts; inclusion of psychiatric comparison groups to establish diagnostic specificity; longitudinal studies to assess treatment response; exploration of brain-peripheral correlations via neuroimaging; and evaluation of S-(PGJ₂)-glutathione’s role in multi-marker diagnostic panels (e.g., alongside markers like bilirubin fractions or uric acid [50]). Additionally, investigating its association with specific symptom dimensions may refine phenotype-biomarker links.

## Conclusions

S-(PGJ₂)-glutathione may represent a preliminary serum biomarker for comorbid depression and anxiety disorders, positioned at the nexus of two core pathophysiological pathways (inflammation and oxidative stress). While internal validation suggests its potential, external validation in large, independent, multi-center cohorts is mandatory to confirm diagnostic specificity, generalizability, and clinical utility before any clinical application can be considered.

## Data Availability

Data in the study could be available from the corresponding author upon reasonable request.

## Declarations

### Declaration of AI and AI-assisted technologies in the writing process

During the preparation of this manuscript, the authors used Doubao (ByteDance, 2026) and DeepSeek-V3.2 for English language polishing, grammatical optimization and academic expression refinement; these AI tools were not applied to generate core research content including study design, data analysis, result interpretation and original conclusions. All authors have thoroughly reviewed, revised and verified the AI-assisted content, and take full responsibility for the accuracy, originality and academic integrity of this manuscript.

## Abbreviations

DAs: Depression and Anxiety Disorders
PGs: Prostaglandins
PGJ₂: Prostaglandins J₂
15d-PGJ₂: 15-deoxy-Δ¹²,¹⁴-prostaglandin J₂
GSH: Glutathione
GSTP1: Glutathione S-transferase P1
HCs: Healthy Controls
LC-MS/MS: Liquid Chromatography-Tandem Mass Spectrometry
ROC: Receiver Operating Characteristic
DCA: Decision Curve Analysis
SBP: Systolic Blood Pressure
DBP: Diastolic Blood Pressure
CRP: C-Reactive Protein
CNS: Central Nervous System
ROS: Reactive Oxygen Species
GST: Glutathione-S-transferase
HAMA: Hamilton Anxiety Rating Scale
HAMD: Hamilton Depression Rating Scale
BMI: Body Mass Index
ESI: Electrospray Ionization Source
OR: Odds Ratio
CI: Confidence Intervals
AUC: Area under the curve
SD: Standard Deviation
PCA: Principal Component Analysis
FC: Fold Change
FDR: False Discovery Rate
MDA: Malondialdehyde
LPO: Lipid Hydroperoxides
SOD: Superoxide Dismutase
Px: GSH-Glutathione Peroxidase
CAT: Catalase
SSRI: Selective Serotonin Reuptake Inhibitors
NOX1: NADPH oxidase 1
